# Fibroblast Growth Factor 21 and Its Association With Oxidative Stress and Lipid Profile in Type 2 Diabetes Mellitus

**DOI:** 10.7759/cureus.17723

**Published:** 2021-09-04

**Authors:** Mudassar Aleem, Hamza Maqsood, Shifa Younus, Ahmed F Zafar, Abdul Subhan Talpur, Hassan Shakeel

**Affiliations:** 1 Internal Medicine, Nishtar Medical University, Multan, PAK; 2 Medicine, Liaquat University of Medical and Health Sciences, Jamshoro, PAK; 3 Neurology, Nishtar Medical University, Multan, PAK

**Keywords:** fibroblast growth factor, type 2 diabetes, oxidative stress, dyslipidemia, lipids

## Abstract

Introduction

Cardiovascular diseases are the leading cause of mortality in diabetic patients. Oxidative stress and mitochondrial dysfunction lead to diabetic cardiomyopathy (DCM) characterized by impaired cardiac structure and function. Hyperglycemia causes oxidative stress, which can lead to microvascular complications, macrovascular complications, and atherosclerosis. Peripheral tissues produce fibroblast growth factor 21 (FGF-21), which has anti-inflammatory properties, increases oxidation of fatty acids, and improves insulin sensitivity. Its increased levels are found in metabolic syndrome and type 2 diabetes mellitus and may also lead to coronary heart disease. Our study sought to measure the serum FGF-21 levels and their associations with lipid profile parameters and oxidative stress in patients with type 2 diabetes mellitus.

Methodology

One-hundred fifty (150) patients of both genders with type 2 diabetes mellitus were recruited along with 150 controls. Simple random sampling was done. After taking relevant history and physical examination, we drew venous blood samples of each patient and sent them to the institutional laboratory for analysis of fasting blood sugar (FBS) levels, glycated hemoglobin (HbA1C), lipid profile, and FGF-21 serum levels. Oxidative stress parameter malondialdehyde (MDA) was estimated and the total antioxidant status by ferric reducing antioxidant power assay (FRAP) was assessed. Patients were followed up after three months to record the glycemic index, and the values were recorded. We used SPSS Software 25.0 (SPSS, Inc., Chicago, USA) to analyze the data. For consideration of results to be statistically significant, a 𝑃 value of < 0.05 was selected.

Results

The levels of serum cholesterol, triglycerides, and low-density lipoprotein (LDL) cholesterol were increased in diabetics compared to controls and were statistically significant (p<0.05). High-density lipoprotein (HDL) cholesterol was lower in diabetic patients as compared to the controls (p<0.05). There was a statistically significant increase in the level of MDA in diabetics compared to controls (p˂0.005). Serum levels of total antioxidant status (FRAP) were decreased in diabetics in comparison with controls (p˂0.005). Serum FGF-21 level was statistically increased in diabetics compared to controls (p˂0.005). FGF-21 and MDA are positively correlated and FGF-21 and FRAP are negatively correlated. Serum FGF-21 is positively correlated with total cholesterol, triglycerides, serum LDL cholesterol, and HDL cholesterol.

Conclusion

Our study concludes that there is a significant correlation between fibroblast growth factor 21, oxidative stress, and abnormal lipid profile in type 2 diabetic patients. FGF-21 could be the target of certain medications used to treat metabolic disorders and obesity.

## Introduction

Diabetes mellitus is a multifactorial disorder characterized by persistent high blood glucose levels associated with aberrations in lipid, carbohydrate, and protein metabolisms leading to water and electrolyte imbalance [1-2]. Cardiovascular diseases are the leading causes of mortality in diabetic patients. Various studies indicate that oxidative stress and mitochondrial dysfunction lead to diabetic cardiomyopathy (DCM) characterized by impaired cardiac structure and function [3-4]. Dyslipidemia is described as high levels of triglycerides, small dense low-density lipoprotein (sdLDL) cholesterol particles, and low levels of high-density lipoprotein (HDL) cholesterol, which is more common in type 2 diabetes mellitus (T2DM). Various factors interplay in the development of dyslipidemia such as visceral fat, insulin resistance, and excessive fatty acids [5]. In type 2 diabetes mellitus (T2DM), chronic hyperglycemia can lead to the generation of reactive oxygen species (ROS), and the ROS-hyperglycaemia interface is involved in the development of the micro-and macrovascular complications of T2DM [3-4].

Imbalance in free radical production and removal alters the cellular metabolism, leading to oxidative stress. Increased intracellular oxidative stress in the body, in turn, is the cause of various chronic diseases [6]. Mitochondrial oxidative phosphorylation, xanthine oxidases, nicotinamide adenine dinucleotide phosphate (NADPH) oxidases, and lipoxygenase reactions can lead to ROS formation [7]. Free radical production is also stimulated by high glucose levels. Hyperglycemia causes oxidative stress, which can lead to microvascular complications, macrovascular complications, and atherosclerosis. Lower antioxidant enzymes due to chronic oxidative stress damage pancreatic β-cells [8].

Peripheral tissues produce fibroblast growth factor 21 (FGF-21), which has anti-inflammatory properties, increases the oxidation of fatty acids, and improves insulin sensitivity. Its increased levels are found in metabolic syndrome and type 2 diabetes mellitus and may also lead to coronary heart disease [9].

A study tells that cultured endothelial cells treated with low-density lipoproteins that were oxidized cause an increase in FGF-21 messenger RNA (mRNA) expression and protein concentration [6]. Its low levels lead to elevated blood insulin, impaired glucose tolerance, and fatty liver. Its deficiency also hastens the progression of diabetic cardiomyopathy. Its vascular protective function is due to adiponectin and sterol regulatory element-binding protein 2 (srebp-2). The quantity and activity of brown adipocytes are inversely related to cardiovascular diseases [10].

Our study sought to measure the serum FGF-21 levels and their associations with lipid profile parameters and oxidative stress in patients with type 2 diabetes mellitus.

## Materials and methods

Study setting

This study was carried out at the inpatient department of Nishtar Medical University and Hospital, Multan.

Subjects, sample size, and sampling technique

One-hundred fifty (150) patients of both genders with type 2 diabetes mellitus were recruited along with 150 controls. Simple random sampling was done. Informed consent was taken before the commencement of the study.

Study design

This research employed a case-control approach to evaluate the association of fibroblast growth factor 21 with oxidative stress and lipid profile in type 2 diabetics.

Inclusion criteria

Patients with type 2 diabetes mellitus taking oral antihyperglycemics with ages ranging between 25 and 60 years were included in this study.

Exclusion criteria

Patients with existing cardiac, liver, and kidney disease or any other acute or chronic disorder were excluded. Patients taking injectable hypoglycemic agents, systemic or inhaled glucocorticoids, or any other medications known to interfere with glucose metabolism and oxidative factors level were also excluded.

Data collection procedure

After taking relevant history and physical examination, we drew venous blood samples of each patient and sent them to the institutional laboratory for analysis of fasting blood sugar (FBS) levels, HbA1C, lipid profile, and FGFR 21 serum levels. Oxidative stress parameter malondialdehyde (MDA) was estimated by the Yagi et al. method [11]. The Benzie et al. method was used to assess the total antioxidant status by ferric reducing antioxidant power assay (FRAP) [12]. Patients were followed up after three months to record the glycemic index, and the values were recorded.

Data analysis

We used SPSS software 25.0 (IBM Corp., Armonk, NY) to analyze the data. We reported the data as means along with standard error. For comparison of values, we employed independent t-tests. Pearson's correlation coefficients were used to determine the correlation. For consideration of results to be statistically significant, a 𝑃 value of <0.05 was selected.

## Results

All patients completed the study. The demographic details and baseline investigation values of the patients of both groups are given in Table 1.

**Table 1:** Demographic and clinical characteristics of the study participants (n=300) HbA1C: glycated hemoglobin

Gender	Patients (n=150)	Controls (n=150)
Males	101 (67.3%)	93 (62%)
Females	49 (32.6%)	57 (38%)
Age Range (Years)		
41-45	19 (12.6%)	20 (13.3%)
46-50	84 (56%)	84 (56%)
51-55	47 (31.3%)	46 (30.6%)
Body mass index (BMI) kg/m^2^​​​​​		
<25	21 (14%)	79 (52.6)
25-30	89 (59.3%)	56 (37.3)
>30	40 (26.6%)	15 (01%)
Waist hip ratio (mean±SD)	1.013±0.03	0.08±0.01
Systolic blood pressure (mean±SD)	129.4±8.2	115.4±6.1
Diastolic blood pressure (mean±SD)	82.7±5.3	76±3.6
Fasting blood sugar (mean±SD)	149.6±29.2	91±11.5
HbA1C (mean±SD)	8.8±0.87	5.3±0.33

The level of serum cholesterol, triglycerides, and LDL cholesterol were increased in diabetics as compared to controls and statistically significant (p<0.05). HDL cholesterol was lower in diabetic patients as compared to the controls (p<0.05) (Table 2).

**Table 2:** Comparison of lipid profile parameters in control and type 2 diabetic patients LDL: low-density lipoprotein; HDL: high-density lipoprotein

Parameters	Patients (n=150)	Controls (n=150)	P-value
Triglycerides (mg/dl)	162.33±23.8	94.8±12.2	0.035
Total cholesterol (mg/dl)	211±29.2	165±9.9	0.041
LDL (mg/dl)	142.5±22.1	102±11.81	0.029
HDL (mg/dl)	29.3±5.7	43.8±7.1	0.033

There was a statistically significant increase in the level of MDA in diabetics compared to control (p˂0.005). Serum levels of total antioxidant status (FRAP) were decreased in diabetics in comparison with controls (p˂0.005). Serum FGF-21 level was statistically increased in diabetics compared to controls (p˂0.005) (Table 3).

**Table 3:** Comparison of FGF-21, oxidative stress marker, and total antioxidant status in control and type 2 diabetic patients MDA: malondialdehyde; FGF-21: fibroblast growth factor 21

Parameters	Patients (n=150)	Controls (n=150)	P value
FGF-21 (pg/ml)	561.4±76.1	239±58.7	0.041
MDA (nmol/l)	11.7±1.4	1.62±0.28	0.034
FRAP (μg/l)	349.4±29.2	615.7±14.78	0.047

FGF-21 and MDA are positively correlated and FGF-21 and FRAP are negatively correlated. Serum FGF-21 is positively correlated with total cholesterol, triglycerides, serum LDL cholesterol, and HDL cholesterol (Table 4).

**Table 4:** Correlation between FGF-21, oxidative stress, total antioxidant status parameters, and lipid profile MDA: malondialdehyde; FGF-21: fibroblast growth factor 21; LDL: low-density lipoprotein; HDL: high-density lipoprotein

Parameters	Pearson correlation coefficient	P-value
FGF-21 Versus		
FRAP	-0.751	0.042
MDA	0.871	0.044
Total cholesterol	0.57	0.032
Triglycerides	0.71	0.035
LDL	0.43	0.029
HDL	0.36	0.026

## Discussion

Obesity raises the risk of a variety of illnesses, including type 2 diabetes mellitus [1]. According to a recent study, Diabetics revealed a higher BMI than controls. Both diabetic and control patients have a higher waist-to-hip ratio (WHR). BMI does not represent body fat distribution while the WHR, as an indicator of central adiposity, is a leading contributor to the development of hypertension, diabetes, dyslipidemia, and insulin resistance [13]. In diabetic patients, serum triglycerides, cholesterol, and LDL levels were all higher than average as compared to the control group [13]. Dyslipidemia is very common in people with type 2 diabetes and leads to a slightly higher risk of coronary heart disease [1]. The key lipid profile alterations in diabetic dyslipidemia are elevated triacylglycerol levels and decreased HDL cholesterol [1].

FGF-21, a novel polypeptide ligand, plays a key role in regulating glucose homeostasis and lipid metabolism. FGF-21 blood levels were higher in mouse models of obesity-related diseases, including chronic hyperglycemia, nonalcoholic fatty liver disease, and atherosclerosis. FGF-21 levels were also higher in the human population with several chronic disorders linked to atherogenic lipid profiles [14].

In our research, FGF-21 levels in the blood were found substantially higher in T2DM patients relative to controls. FGF-21 had a positive correlation with triglycerides, total cholesterol, and LDL cholesterol but had a negative correlation with HDL cholesterol. High serum FGF-21 levels were positively linked to metabolic diseases such as diabetes, obesity, mitochondrial disease, and aging [15]. According to several studies, FGF-21 stimulates fatty acids oxidation, the production of ketone bodies, and the inhibition of lipogenesis. FGF-21 controls glucose and lipid metabolism and has thus been identified as a potential therapeutic target for metabolic disease [16].

Recent studies have reported that rFGF21 therapy depresses the serum amounts of cholesterol, LDL, triglyceride, and free fatty acid (FFA) thus increasing high-density lipoprotein (HDL) and lowering body weight [17]. FGF-21 levels in the blood are higher in people with reduced glucose tolerance and diabetes, as reported in previous literature. Recent research has also discovered that the FGFR1/klotho/Akt signaling cascade in cardiomyocytes prevents myocardial damage and apoptosis following ischemia-reperfusion [18].

MDA is produced when the carbon chain of unsaturated fatty acids is ruptured during lipid peroxidation [10]. In our research, diabetics had higher MDA levels than controls, which was statistically significant (p<0.001). In diabetics, overall antioxidant status (FRAP) was lower than in controls (p<0.001). FGF-21 was shown to have a significant correlation with thiobarbituric acid reactive substances (TBARS) (r=0.875, p<0.01) and a negative correlation with FRAP (r=-0.867, p<0.01).

One of the most significant pathogenesis of atherosclerosis is oxidative stress. Oxidative stress generates ROS and downregulates the innate antioxidant protection mechanisms of the body [8]. According to studies, FGF-21 has a role in the prevention of atherosclerosis [10]. FGF-21 decreases oxidative stress in cardiomyocytes and prevents injury by stimulating antioxidative pathways [19]. Based on the findings of our study, we suggest the consumption of diet including probiotics/synbiotics, paleolithic diet, and vegetable, and seed oils. The addition of these diets to the daily routine can lead to decreased oxidative stress in various chronic diseases including diabetes mellitus [20-21].

Limitations

Our study has a few limitations as well. First, we could not establish the association of FGFR21 levels with the severity of diabetes mellitus based on HbA1C. Second, the high levels of FGF-21 found in our study could be compensatory responses to maintain metabolic homeostasis. Further longitudinal studies are needed to explore the role of FGF-21 as a novel marker in predicting cardiovascular risk in patients with chronic diseases, especially diabetes mellitus.

## Conclusions

Our study concludes that there is a significant correlation between fibroblast growth factor 21 (FGF-21), oxidative stress, and abnormal lipid profile in type 2 diabetic patients. FGF-21 could be the target of certain medications used to treat metabolic disorders and obesity.
